# Exploring the Impact of Vascular Alignment and Grafting on Grapevine Physiology and Growth

**DOI:** 10.1111/ppl.70704

**Published:** 2025-12-28

**Authors:** Ana Villa‐Llop, Ignacio Buesa, Maider Velaz, Maite Loidi, José Mariano Escalona, Antoni Sabater, Luis Gonzaga Santesteban, Nazareth Torres

**Affiliations:** ^1^ Vitis Navarra Nursery Larraga Navarra Spain; ^2^ Institute for Multidisciplinary Research in Applied Biology (IMAB‐UPNA) Public University of Navarre, Campus Arrosadia Pamplona Spain; ^3^ Departament of Agronomy, Biotechnology and Food Science Public University of Navarre Pamplona Navarra Spain; ^4^ Department of Ecology and Global Change Desertification Research Centre‐CIDE, CSIC‐UV‐GVA Moncada Spain; ^5^ Research Group on Plant Biology and Agroecology (PlantAgro) ‐ Department of Biology University of the Balearic Islands (UIB) Palma Balearic Islands Spain; ^6^ Agro‐Environmental and Water Economics Institute‐University of Balearic Islands (INAGEA‐UIB) Palma Balearic Islands Spain

**Keywords:** cambium, drought, rootstock‐scion interaction, stomatal conductance, water potential

## Abstract

Grafting has been fundamental in viticulture since the phylloxera crisis of the late 19th century; nevertheless, the functional consequences of vascular connection on the graft union remain poorly understood. The effects of grafting on *Vitis vinifera* cv. Tempranillo (Te) were evaluated using two complementary approaches: (1) cambial alignment, comparing completely aligned (CA) versus partially aligned (PA) unions; and (2) grafting and scion–rootstock interaction, comparing heterografts (Te/110R and Te/RG8), homografts (Te/Te), and ungrafted Te cuttings. These approaches were tested through three experiments: a vineyard trial and two pot trials under well‐watered (WW), moderate water stress (MWS), and recovery (R) regimes. In the vineyard, CA plants exhibited greater vegetative growth and gas exchange, particularly on 110R, whereas the vigorous RG8 rootstock mitigated the effects of misalignment. Under MWS conditions, CA adopted a drought‐avoidant strategy with earlier stomatal closure and higher root allocation, whereas PA maintained higher stomatal conductance, recovered photosynthesis faster after rewatering, and prioritised shoot and rootstock growth, especially on RG8. Finally, grafted plants were more sensitive to water stress than ungrafted plants, while homografts accumulated the greatest biomass and root investment, suggesting more efficient vascular connectivity compared with heterografts. Our study highlights that cambial alignment, grafting, and partner interactions influence plant development and physiological performance; however, long‐term studies are needed to clarify how vascular connectivity at the graft union affects transport processes, stress responses, and ultimately vine longevity under different scion–rootstock combinations.

## 1 Introduction

Grafting became a mandatory practice in viticulture after the accidental introduction of phylloxera (*Daktulosphaira vitifoliae* Fitch) in Europe at the end of the 19th century. It combines the desired traits of the *Vitis vinifera* L. scions with the disease resistance and environmental adaptability of rootstocks of American *Vitis* species or their hybrids (Ollat et al. 2016). Then, grafting evolved from several techniques to predominantly bench grafting using the omega‐style, which is the most employed today due to its higher success rate (Mary et al. 2017). Despite the benefits of grafting to combat phylloxera being widely recognised, debate has surrounded this practice from the very beginning. As early as 1908, only 25 years after grafting became widespread in France, the French botanist Lucien L. Daniel raised concerns that grafted vines would not reach the longevity of ungrafted vineyards, ageing faster and requiring periodic renewal (Daniel 1908). He also described anatomical irregularities at the graft union, where the bourrelet (thickened ridge at the graft union) caused deviations in the vascular tissues, reduced the number of conducting vessels, and acted as a barrier to sap flow. Despite these early concerns, little research has been conducted in viticulture to better understand the implications of grafting on grapevine performance. Fortunately, in the last decade, there have been significant contributions in terms of characterising vascular connections (Bahar et al. 2010; Camboué et al. 2024, 2025; Carrere et al. 2022; Janoueix et al. 2024; Milien et al. 2012; Spilmont et al. 2021), anatomical, biochemical and genetic interactions between scion and rootstock (Assunção et al. 2019; Canas et al. 2015; Cookson et al. 2019; Loupit et al. 2022; Loupit and Cookson 2020), agronomic performance at the nursery (Çelik 2000; Korkutal et al. 2013; Köse et al. 2015; Marín et al. 2018, 2022; Villa‐Llop et al. 2025) and at the vineyard level (Korkutal et al. 2018; Marín et al. 2022; Buesa et al. 2023; Villa‐Llop et al. 2025). Nevertheless, there is still little information on how the vascular connections at the graft union condition the ecophysiological response of grapevines. In this regard, two approaches can be used to modify the connections at the graft union: (1) altering the contact area between the scion and the rootstock at the moment of grafting through partial alignment of the cuttings grafted (Marín et al. 2022), or (2) comparing normally grafted plants (heterografts) with homografts (rootstock and scion of the same genotype) and with ungrafted cuttings as a reference control (Cookson et al. 2013; Tedesco et al. 2020, 2021).

Regarding the first approach, the lack of alignment between the scion and the rootstock is recognised as a critical factor contributing to graft failure (Bester 2020), which limits vascular connections (Milien et al. 2012) and restricts xylem and phloem translocation (Shtein et al. 2017). Marín et al. (2022) examined the effect of rootstock‐scion alignment on plant physiology and water use under field and controlled conditions, comparing omega‐grafted plants with complete alignment (CA) of the scion and rootstock cuttings to those with partial alignment (PA). They demonstrated that alignment influenced early plant growth and physiology, with CA plants exhibiting greater vegetative growth and higher stomatal conductance during the first year, particularly under controlled conditions. However, no significant differences were observed between CA and PA plants from the second year onward in the field, suggesting that the effect diminished over time. Therefore, mid‐term effects of CA and PA alignment on vine growth and physiology have not yet been systematically evaluated.

Regarding the second approach, homografts and ungrafted controls have proven to be valuable tools to separate intrinsic effects of grafting from those arising from rootstock–scion interactions. Clemente Moreno et al. (2014) and Cookson et al. (2013) used homografts to investigate transcriptional and histological responses during graft union formation, revealing changes related to cell wall modification, hormone signalling, and wound responses. More recently, Tedesco et al. (2020, 2021, 2023) extended this approach to metabolomic studies, comparing homografts, heterografts, and ungrafted cuttings. However, none of these studies performed ecophysiological measurements to assess the implications of the graft union on plant behaviour.

The present study aims to better understand the relevance of grafting on plant development, gas exchange, and water relations through three complementary experiments: (1) Experiment 1, an adult field trial to compare heterografted plants with two degrees of graft alignment (CA vs. PA); (2) Experiment 2, a pot trial with young plants to assess the impact of graft alignment under controlled conditions with three irrigation regimes, well‐watered (WW), moderate water stress (MWS), and recovery (R); and (3) Experiment 3, a pot trial with young plants where heterografts were compared to homografts and ungrafted cuttings under the different water regimes.

## 2 Materials and Methods

### 2.1 Plant Material Preparation

All the experiments required the preparation of grafted plants produced ad hoc years before physiological measurements, which were obtained from the Vitis Navarra nursery (Larraga, Navarra, Spain). Tempranillo cv. ( *V. vinifera* L.) was used as scion and grafted onto two rootstocks: 110 Richter ( *V. berlandieri* × *V. rupestris* ) and RG8 (41B × 110R). Tempranillo is the most widely planted red grapevine cultivar in Spain, while 110R is the predominant rootstock in national grapevine nurseries (Marín et al. 2019). RG8 is a recently authorised commercial rootstock developed by Vitis Navarra S.A.T., which is increasingly used in scientific studies to assess its physiological performance (Alonso‐Forn et al. 2025; Buesa et al. 2023; Flor et al. 2025; Marín et al. 2022). Grafting, rooting, and production processes were managed according to commercial practices, as described in Marín et al. (2022) and Villa‐Llop et al. (2025).

In short, dormant hardwood of the scion and rootstock was collected in winter from certified free‐virus mother fields located in Larraga, Navarra (42°27′45″ N, 1°48′13″ W, 325 m a.s.l.). Until grafting, single‐bud cuttings of the scion and manually de‐budded rootstock cuttings (38–40 cm) were stored at 4°C and 85%–90% relative humidity inside closed plastic bags. Before grafting, all materials were disinfected and soaked in water at room temperature for 24 h to facilitate rehydration. Omega grafting was conducted in early spring using an Omega Star foot‐drive machine (Wahler OMEGA GmbH & Co. KG). Graft unions were waxed, and the grafted plants were placed in boxes in a stratification chamber at 20°C–25°C and 90%–95% relative humidity for approximately 20 days. Following successful callusing, graft bases were dipped in rooting hormone, graft union re‐waxed, and transferred to the nursery field. After one growing season and once leaf fall occurred, grafted plants were dug up and subjected to strict quality control.

#### 2.1.1 Obtention of Plants Differing in Rootstock‐Scion Alignment

**FIGURE 1:**
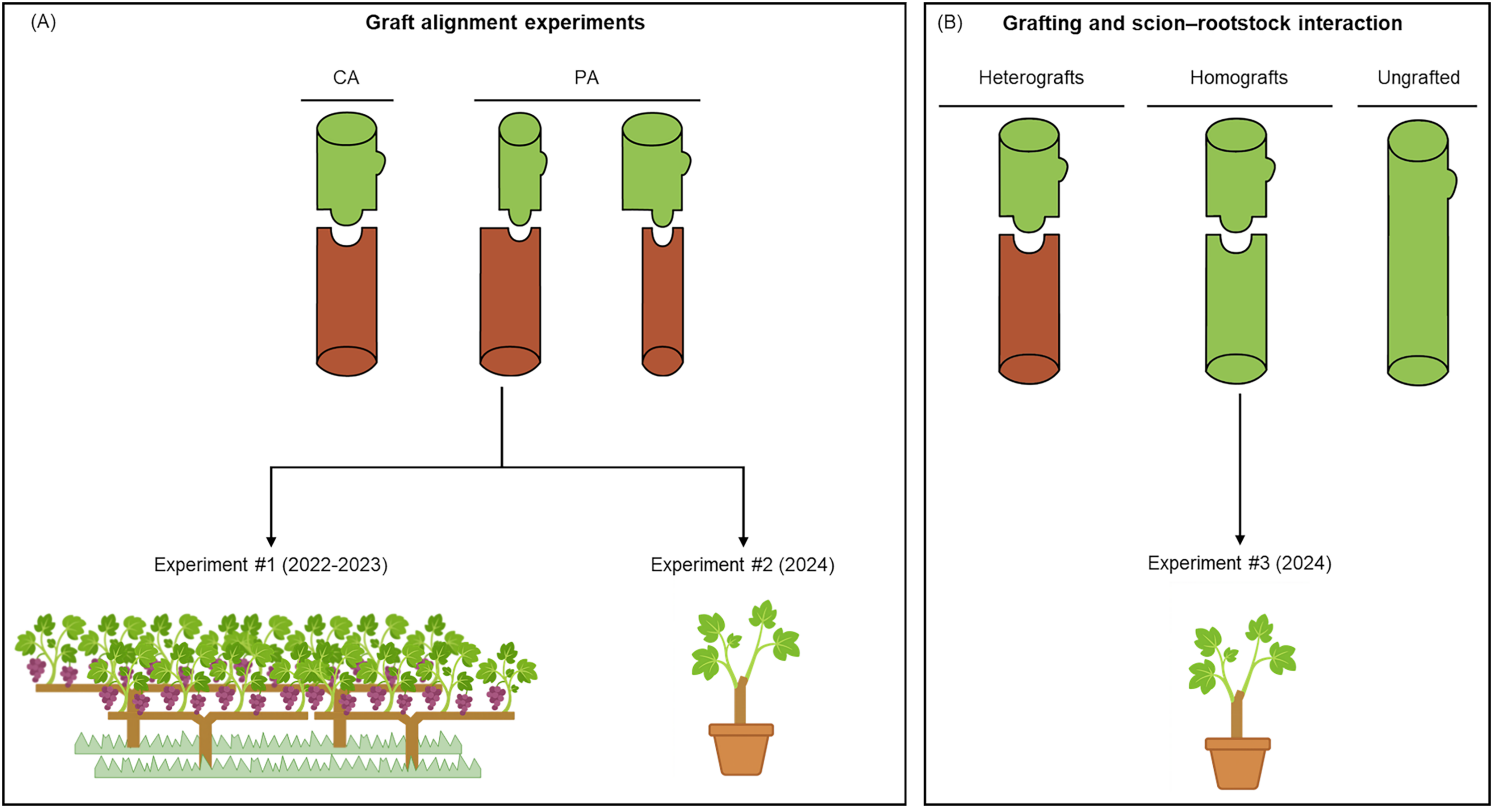
Schematic summary of the experimental design. (A) illustrates the graft alignment experiments comparing complete alignment (CA) and partial alignment (PA) between scion and rootstock cuttings, conducted under field conditions (Experiment #1, 2022–2023) and in potted plants (Experiment #2, 2024). (B) illustrates the scion–rootstock interaction experiment conducted under potted conditions (Experiment #3, 2024), including heterografted, homografted and ungrafted plants. Experiments #2 and #3 were carried out under three watering regimes: Well‐watered, moderate water stress and recovery.

Regarding the experiments that used completely and partially aligned (CA and PA) omega‐grafted plants (Experiments 1 and 2). CA omega‐grafted plants were obtained using scion and rootstock cuttings of similar diameters, whereas PA omega‐grafted plants were grafted with deliberate cutting diameter mismatch, ensuring partial cambium contact (Figure 1A). Tempranillo cv. ( *V. vinifera* L.) was used as scion, and 110 Richter ( *V. berlandieri* × *V. rupestris* ) and RG8 (41B × 110R) as rootstocks.

#### 2.1.2 Obtention of Heterografts, Homografts, and Ungrafted Cuttings

For Experiment 3, four types of plants were prepared: Heterografts of Tempranillo cv. grafted onto 110R and RG8 (Te/110R and Te/RG8), Tempranillo homografts (Te/Te; one‐bud scion of Tempranillo grafted onto manually de‐budded Tempranillo cuttings of 38–40 cm) and ungrafted‐unrooted Tempranillo cuttings of the same total length, with only one bud left at the apical end (Te) (Figure 1B).

### 2.2 Experimental Designs

#### 2.2.1 Experiment #1: Grafting Alignment Implications Under Field Conditions

The first experiment was established in 2018 in Murieta, Navarra (42°39′49″ N, 2°09′27″ W, elevation 500 m), within a commercial vineyard belonging to Quaderna Via winery. Grapevine plants, grafted in 2017 with the two degrees of alignment (CA and PA), were planted following a completely randomised design with three blocks. Each block included 10 plants per rootstock and grafting alignment, totalling 30 plants per treatment (up to 120 experimental vines). Planting density was 2777 plants per hectare (3 × 1.2 m); plants were trained to a bilateral cordon Royat under vertical shoot positioning. Vineyard management, including pruning, pest control, and fertilisation, was conducted by the winery using conventional practices appropriate for the area, including deficit drip irrigation. Three irrigation events of 12 h each were applied per season, approximately 150 L per plant. The soil is classified as glacis‐type quaternary sedimentary, with a loamy texture, highly calcareous content (9% active lime), and organic matter content around 2%. Local climate is Continental‐Mediterranean, characterised by warm, dry summers and cool winters. Weather data for the past decade, as well as for the experimental period, 2021 to 2023, was obtained from the Agroclimatic Information Service (SIAR) station located in Ancín, near the vineyard (42°39′20.5″ N, 2°10′18.8″ W, 469 m a.s.l.).

Ecophysiological and agronomic characterisation was conducted over three consecutive growing seasons, 2021, 2022, and 2023, corresponding to the fourth, fifth, and sixth years of plant development. Prior to the start of measurements, six uniform plants per treatment (*n* = 6), two per block, were selected based on trunk cross‐sectional area (TCSA) for carrying out physiological and agronomic assessments. TCSA was determined using two orthogonal diameter measurements taken above the graft union with a digital calliper (Mitutoyo CD67‐S15PP).

#### 2.2.2 Experiment # 2: Grafting Alignment Implications Under Controlled Conditions

The experiment was established in May 2022 at the Public University of Navarra experimental farm in Pamplona, Spain (42°47′36″ N, 1°37′52″ W, 430 m elevation), using plants grafted in 2021. Twelve plants per grafting combination were planted in 20 L pots filled with a mixture of vermiculite, sand, and commercial substrate, arranged in a completely randomised design. In winter 2023, all plants were pruned to one spur with two nodes. Each pot was equipped with a pressure‐compensated emitter to ensure uniform water application.

During the first year of measurements (2023), pots were maintained well‐irrigated, applying drip irrigation twice daily for 10 min (2.3 L h^−1^ per plant). In 2024, in order to evaluate the implications of graft alignment under different water regimes, drip irrigation was applied as in the previous season until the 3rd of June 2024, when irrigation was suspended to induce moderate water stress and was resumed on 1st of July 2024, once mean stem water potential (*Ψ*_stem_) and stomatal conductance (*g*_s_) dropped below −0.8 MPa and 0.05 mol H_2_O m^−2^ s^−1^, respectively. These thresholds correspond to mild to moderate water deficits, as defined by Deloire et al. (2020) and Flexas et al. (2002).

Based on *Ψ*_stem_ and *g*_s_ dynamics, three water regimes were defined: (1) well‐watered (WW) from June 3rd to June 10th, when both *Ψ*_stem_ and *g*_s_ mean values began to decline; (2) moderate water stress (MWS) from June 10th to July 1st, during which *Ψ*_stem_ decreased progressively and *g*_s_ reached minimum mean values; and (3) recovery (R) from July 1st to July 15th, during which *Ψ*_stem_ and *g*_s_ increased following rewatering. Four plants per treatment were monitored throughout the experiment for physiological and vegetative parameters, and final measurements of growth and biomass allocation were recorded in October 2024 (*n* = 4).

#### 2.2.3 Experiment # 3: Comparison of Heterografts, Homografts, and Ungrafted Cuttings

Following the same timeline as in Experiment 2, in May 2022, Tempranillo homografts, Tempranillo/110R and Tempranillo/RG8 heterografts, and Tempranillo ungrafted‐unrooted cuttings were planted in 20 L pots at the Public University of Navarra experimental farm in Pamplona, Spain (42°47′36″ N, 1°37′52″ W, 430 m elevation). The experiment followed a completely randomised design, with 12 plants per combination. As in Experiment 2, four plants per treatment were monitored throughout the experiment (*n* = 4). Evaluation began in 2023 under fully watered conditions. In 2024, water was cut off on June 3rd and resumed on July 1st.

### 2.3 Physiological and Agronomic Determinations

#### 2.3.1 Water Relations

Pre‐dawn leaf water potential (*Ψ*_pd_) and stem water potential (*Ψ*_stem_) were assessed on healthy leaves (*n* = 6 in the vineyard experiment; *n* = 4 in the pot experiment), using a pressure chamber (Soil Moisture Equipment Corp.). *Ψ*_pd_ was measured with readings taken between 3:00 and 5:00 am solar time. Field readings were recorded on the 12th of August 2022 and 18th of August 2023, whereas a single measurement was taken on August 24, 2023, in Experiment 2. Each measurement of *Ψ*_stem_ was taken at mid‐morning (9:30 to 11:00 am solar time), with leaves bagged 1 h prior to measurement using reflective metallised polyethylene bags (SonocoRF, Sonoco Products Co.). These measurements were recorded on August 12th in the field, while during the moderate stress trial, *Ψ*_stem_ measurements were conducted every 3 days from June 3rd to July 15th, 2024, in the pot experiments.

#### 2.3.2 Leaf Gas Exchange Measurements

Leaf gas exchange measurements in the field were performed with an infrared open gas exchange analyzer (Li‐6400xt, Licor Inc.), equipped with a 6 cm^2^ cuvette. In the pot experiment, measurements were taken using a portable photosynthesis system (TARGAS‐1, PP Systems) with leaves fully covering a 4.5 cm^2^ cuvette. Photosynthetic rate (*A*_N_), stomatal conductance (*g*_s_), and leaf transpiration (*E*) were measured per unit leaf area, with measurements taken under steady‐state conditions with fully sun‐exposed, photosynthetically active leaves (PAR > 1500 μmol m^−2^ s^−1^) at 400 ppm of CO_2_ concentration.

In the vineyard, measurements were taken on August 12, 2022, and August 18, 2023, between 8:30 and 11:30 a.m. solar time. In pots, a single measurement was conducted on August 24, 2023, to evaluate the effect of graft alignment under non‐stress conditions. Then, the water‐stress experiment was carried out in 2024, with data recorded every 3 days between 10:00 and 11:30 h solar time from June 3rd to July 15th, coinciding with *Ψ*_stem_ assessments.

To integrate the temporal dynamics of plant water status and leaf gas exchange in the pot experiments, the area under the curve (AUC) was calculated for each parameter using natural cubic splines (Friedman 1991) across the three water regimes (WW, MWS, and R). To enable comparison between regimes, AUC values were normalised by dividing by the number of days between the first and the last measurement within each regime (Torres et al. 2021).

#### 2.3.3 Vegetative Growth Assessment and Yield Determination

Vegetative growth of each experimental plant was assessed before winter pruning, using a digital calliper (Mitutoyo CD67‐S15PP, Kanagawa, Japan). TCSA was estimated by measuring the two orthogonal diameters above the graft junction, at approximately 25 cm from the soil. The number of shoots per plant was recorded (Nb. S), and two orthogonal diameter measurements per shoot were taken at the middle part of the first internode. These values were used to calculate the mean and total shoot cross‐sectional area (SCSA) for each plant.

In Experiment 1, vineyard performance at harvest was assessed on 10 plants per combination. Measurements included the total number of clusters (No C) and yield (Y), determined using a field dynamometer (ILSA 9702).

Berry composition was assessed at each treatment using a 200‐berry sample collected one day before harvest from 10 plants per treatment block. Berry samples were weighed using a precision balance (Quintix, Sartorius) to determine mean berry weight (BW). Potential alcohol (PA) expressed as ° and pH were measured by Fourier‐transform infrared (FTIR) spectroscopy using a Foss Winescan Flex (FOSS). The mean cluster weight (CW) was calculated as the ratio of yield to cluster number. Harvest was carried out on September 24th, 15th, and 28th in 2021, 2022, and 2023, respectively.

#### 2.3.4 Biomass Determinations in Pot Experiments

In October 2024, four plants per treatment were carefully removed from their pots for biomass evaluation. Roots were separated from the substrate by manual washing. Plants were separated into three parts: roots, trunks, and shoots. Each part was freshly weighed using a portable precision balance (Ohaus, Ohaus Corp.). All samples were then oven‐dried at 70°C (Conterm, JP Selecta) to constant weight to quantify dry biomass accumulation.

### 2.4 Statistical Analysis

Statistical analyses were performed using RStudio software (version 1.4.1103; RStudio Team 2021). Data normality and homoscedasticity were tested using the Kolmogorov–Smirnov and Levene tests, respectively.

For experiment 1 (*n* = 6), a three‐way analysis of variance (ANOVA) was performed to analyze the effects of year (Y), rootstock (R), and grafting alignment (GA). Since no significant Y × R × GA interactions were detected, a two‐way ANOVA (R × GA) was conducted. The *g*_s_—*Ψ*_stem_ regressions for each graft alignment–rootstock combination were analyzed using a two‐way analysis of covariance (ANCOVA), with *Ψ*_stem_ as a covariate (*p* < 0.05).

A two‐way ANOVA was used to evaluate the effect of graft alignment and rootstock on all measured variables in experiment 2 (*n* = 4), while a one‐way ANOVA was conducted in experiment 3 (*n* = 4).

For all analysis, means ± standard errors (SE) were calculated. When the *F*‐value was significant (*p* ≤ 0.05), post hoc comparisons were performed using Duncan's honest significance difference (HSD) test, which was executed using ‘emmeans’ (1.7.1–1) R packages (Lenth et al. 2025).

## 3 Results

### 3.1 Experiment #1: Grafting Alignment Implications Under Field Conditions

The meteorological conditions at the experimental field from January to December are shown in Figure S1. The 10‐year averages were 568 mm annual rainfall and 1672°C GDD from April to October. The 2021 season was cooler and wetter than the following 2 years, with 588 mm of rainfall and 1377°C GDD. The 2022 season was the driest and warmest of the past decade, with only 375 mm of annual rainfall, less than 130 mm during the vegetative period, and a heat accumulation of 1843°C GDD, significantly above the long‐term mean. In contrast, 2023 was cooler and wetter than 2022, recording 697 mm of rainfall and 1672°C GDD, both above the decadal averages.

**FIGURE 2:**
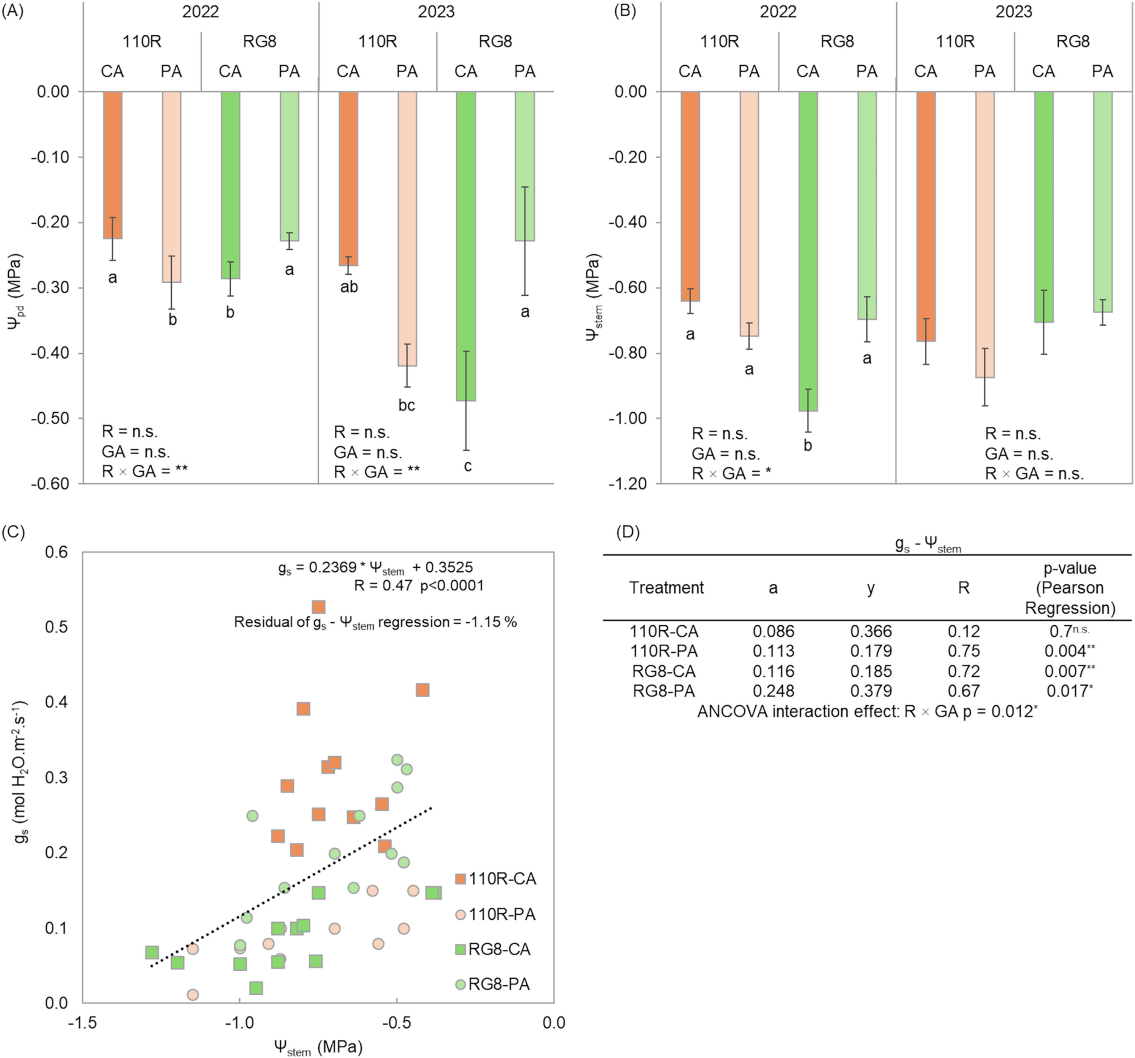
Effect of rootstock (R; 110R and RG8) and graft alignment (GA; CA: Completely aligned and PA: Partially aligned) on (A) pre‐dawn, (B) stem water potential, (C) *g*_s_ and *Ψ*_stem_ relationship and (D) the individual linear regression equation for each rootstock‐alignment combination, measured in 2022 and 2023. Each bar represents the mean value (*n* = 6) ± SE in (A) and (B) figures. Different letters indicate significant differences (*p* ≤ 0.05) for the R × GA interaction, according to two‐way ANOVA followed by Duncan's new multiple range test. n.s., *, **, and *** indicate non‐significant and significance at 5%, 1%, and 0.1% probability levels, respectively. “*a*” indicates the slope of the regression and “*y*” the intercept, “*R*” the correlation coefficients and “*p*‐value” the significance of the regressions according to Pearson analysis.

Daily plant water status, assessed by *Ψ*_pd_ and *Ψ*_stem_, varied across graft alignments and rootstocks (Figure 2). For *Ψ*_pd_ values (Figure 2A), the pattern on 110R was consistent across years, with CA plants showing less negative values than PA plants. In 2022, *Ψ*_pd_ averaged −0.23 and −0.29 MPa in CA and PA plants, respectively. While in 2023 the difference was wider, −0.27 and −0.42 MPa, respectively. On RG8, the trend was reversed in both years, with PA plants maintaining less negative *Ψ*_pd_ values than CA plants. In 2022, PA plants reached −0.23 MPa compared with −0.29 MPa in CA, and in 2023, the contrast was larger −0.23 MPa in PA and −0.47 MPa in CA plants.

For *Ψ*_stem_ values (Figure 2B), differences between graft alignment and rootstock were smaller and less consistent. On 110R, CA plants showed slightly higher *Ψ*_stem_ values than PA plants in both years: −0.64 MPa and −0.76 in 2022, and −0.75 and −0.87 MPa in 2023. On RG8, the pattern varied: in 2022, PA plants maintained higher *Ψ*_stem_ (−0.70 MPa) compared with CA plants (−0.98 MPa), whereas in 2023 the values were similar between graft alignments (−0.71 MPa for CA and −0.68 MPa for PA).

The range of water potential recorded across treatments allowed the evaluation of stomatal responses through the relationship between *g*_s_ and *Ψ*_stem_, showing an overall moderate correlation (Figure 2C), with *g*_s_ decreasing as *Ψ*_stem_ became more negative. The interaction between graft alignment and rootstock was significant, indicating that the relationship between *g*_s_ and *Ψ*_stem_ was influenced by the combination of both factors.

When analyzing by treatment, the relationship between *g*_s_ and *Ψ*_stem_ was significant for all combinations except 110R‐CA (Figure 2D). The highest slope (a) of the *g*_s_—*Ψ*_stem_ regression was observed in the RG8 combinations (0.248 for PA and 0.116 for CA), whereas 110R‐CA showed the lowest slope (0.086). The residuals of the *g*_s_—*Ψ*_stem_ regression indicated a slight underestimation (−1.15%), suggesting some variability in stomatal behaviour not totally explained by *Ψ*_stem_.

Rootstock genotype and graft alignment showed a significant interaction for *A*_N_, g_s_, and *E*, but not for VPD, indicating that the effect of alignment was modulated by the rootstock (Table 1).

**TABLE 1:** Effect of rootstock (R; 110R and RG8) and graft alignment (GA; CA: Completely aligned and PA: Partially aligned) on stomatal conductance (*g*_s_), net photosynthesis (*A*_N_), evapotranspiration (*E*), and vapour pressure deficit (VPD) (Experiment #1).

	Rootstock	Graft alignment	*g*_s_ (mol HO_2_ m^−2^ s^−1^)	*A*_N_ (μmol CO_2_ m^−2^ s^−1^)	*E* (mol H_2_O m^−2^ s^−1^)	VPD (kPa)
2022	110R	CA	0.29 ± 0.03a	13.0 ± 1.4	8.2 ± 0.7	3.2 ± 0.1
		PA	0.09 ± 0.01c	11.7 ± 1.0	6.2 ± 0.8	3.8 ± 0.2
	RG8	CA	0.08 ± 0.01c	11.3 ± 1.3	6.4 ± 1.0	3.6 ± 0.2
		PA	0.17 ± 0.01b	11.7 ± 0.9	6.9 ± 0.7	3.7 ± 0.2
		R	**	n.s.	n.s.	n.s.
		GA	**	n.s.	n.s.	n.s.
		R × GA	***	n.s.	n.s.	n.s.
2023	110R	CA	0.32 ± 0.05a	14.8 ± 1.6a	4.9 ± 0.7ab	2.0 ± 0.1
		PA	0.09 ± 0.02b	11.0 ± 1.5ab	3.0 ± 0.6b	2.5 ± 0.2
	RG8	CA	0.10 ± 0.02b	9.8 ± 0.9b	3.1 ± 0.6b	2.3 ± 0.2
		PA	0.25 ± 0.04a	14.1 ± 1.6ab	5.1 ± 0.6a	2.3 ± 0.3
		R	n.s.	n.s.	n.s.	n.s.
		GA	n.s.	n.s.	n.s.	n.s.
		R × GA	***	**	**	n.s.

*Note:* Mean value (*n* = 6) ± SE. Different letters indicate significant differences (*p* ≤ 0.05) between R and GA, according to two‐way ANOVA followed by Duncan's new multiple range test. n.s., **, and *** indicate non‐significant, significance at 1%, and 0.1% probability levels, respectively.

In 110R, CA plants showed the highest *g*_s_ values (0.29 and 0.32 mol H_2_O m^−2^ s^−1^), which supported greater *A*_N_ and *E* rates (13 and 14.8 μmol CO_2_ m^−2^ s^−1^; 8.2 and 4.9 mmol H_2_O m^−2^ s^−1^, respectively). In contrast, PA exhibited lower *g*_s_ (0.09 mol H_2_O m^−2^ s^−1^), accompanied by reduced *E* (6.2 and 3 mmol H_2_O m^−2^ s^−1^) and a moderate decline in *A*_N_ (11 μmol CO_2_ m^−2^ s^−1^). In RG8, CA plants showed a similar pattern to 110R‐PA plants, with low *g*_s_ (0.08 and 0.10 mol H_2_O m^−2^ s^−1^), reduced *E* (6.4 and 3.1 mmol H_2_O m^−2^ s^−1^, respectively), and comparable *A*_N_ (11.3 and 9.8 μmol CO_2_ m^−2^ s^−1^). In contrast, RG8‐PA exhibited higher *g*_s_ (0.17 and 0.25 mol H_2_O‧m^−2^‧s^−1^), which supported greater *E* (6.9 and 5.1 mmol H_2_O m^−2^ s^−1^) and higher *A*_N_ (11.7 and 14.1 μmol CO_2_ m^−2^ s^−1^).

Regarding vegetative growth, graft alignment influenced cumulative trunk growth and shoot development; this effect being modulated by rootstock (Table 2). Graft alignment significantly affected TCSA, with CA plants exhibiting higher cumulative TCSA values than PA plants across rootstocks (8.1 and 7.1 cm^2^, respectively). The effect was particularly evident on RG8, where CA plants reached 11.5 versus 9.3 cm^2^ in PA plants. The interaction between graft alignment and rootstock influenced cumulative SCSA. CA plants grafted onto 110R showed the highest values, outperforming PA plants (126.1 and 108.4 cm^2^, respectively). According to our results, this response appears to be modulated by rootstock, as differences between CA and PA were less consistent on RG8 (111.4 and 123.7 cm^2^, respectively).

**TABLE 2:** Effect of rootstock (R; 110R and RG8) and graft alignment (GA; CA: Completely aligned and PA: Partially aligned) on cumulative trunk and cross‐sectional area (TCSA, SCSA) from 2021 to 2023 (Experiment #1).

Rootstock	Graft alignment	TCSA (cm^2^)	SCSA (cm^2^)
110R	CA	8.7 ± 0.2	126.1 ± 1.6a
	PA	8.0 ± 0.6	108.4 ± 1.9b
RG8	CA	11.5 ± 1.1	111.4 ± 2.4ab
	PA	9.3 ± 0.9	123.7 ± 2.6ab
	R	***	n.s.
	GA	*	n.s.
	R × GA	n.s.	**

*Note:* Mean value (*n* = 6) ± SE. Different letters indicate significant differences (*p* ≤ 0.05) between R and GA, according to two‐way ANOVA followed by Duncan's new multiple range test. n.s., *, **, and *** indicate non‐significant, significance at 5%, 1%, and 0.1% probability levels, respectively.

Graft alignment between scion and rootstock did not influence any parameter measured related to yield performance and berry composition (Table S1). The vineyard was planted in 2018, and 2021 was the first season expected to reach regular production. Mean Y increased from 1.62 kg plant^−1^ in 2021 to 3.58 kg plant^−1^ in 2022, before declining to 1.55 kg plant^−1^ in 2023. The decline in 2023 across all treatments likely reflected a vintage effect associated with the high production load of 2022.

The cluster number followed a similar trend, increasing from a mean of 10.8 clusters per plant in 2021 to 16.9 in 2022, and then declining to 12.8 in 2023 (Table S1). Cluster and berry weight varied mainly between years, with the highest cluster weights in 2022, which ranged from 194 g (110R‐CA) to 232 g (RG8‐PA), and the lowest in 2023, from 103 g (110R‐CA) to 127 g (RG8‐PA). Berry weight remained stable across seasons, ranging from 1.5 to 2.0 g. Potential alcohol (PA) levels were consistent across treatments, with year‐to‐year variation likely related to crop load and ripening conditions. Values were higher in 2021 (13.8°–14.5°) and lower in 2022 (12.9°–13.4°). Must pH showed little variation, ranging from 3.42 to 3.75, with only minor differences between rootstocks in some seasons (Table S1).

### 3.2 Experiment # 2: Grafting Alignment Implications Under Controlled Conditions

In 2023, neither rootstock, graft alignment, nor their interaction affected plant water status or photosynthesis: *Ψ*_pd_, *Ψ*_stem_, *g*_s_, and *A*_N_ showed no significant differences among treatments (Table S2).

**FIGURE 3:**
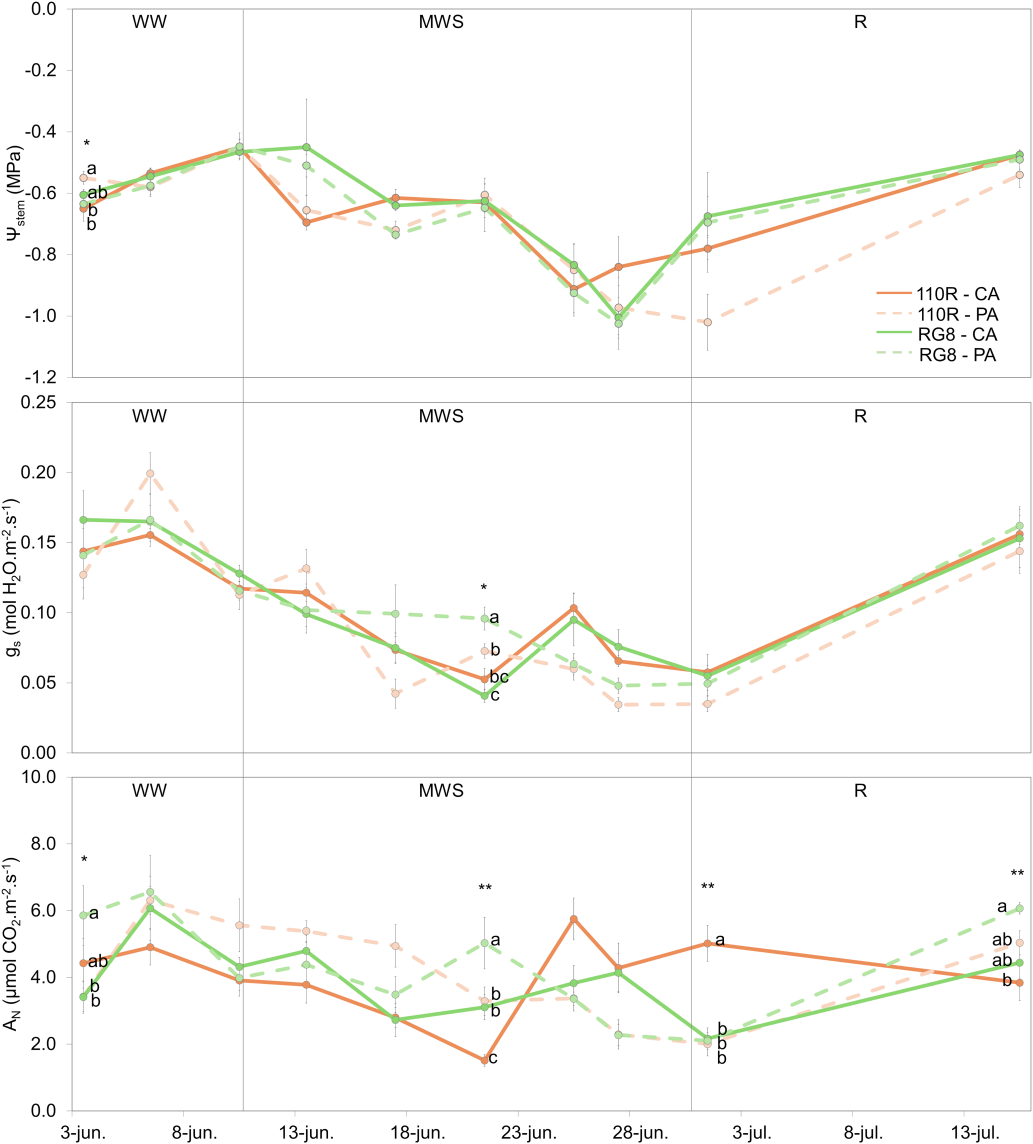
Effect of the rootstock (110R and RG8) and the grafting alignment (CA and PA) on water potential (*Ψ*_stem_), leaf stomatal conductance (*g*_s_) and leaf net carbon assimilation (*A*_N_), under three water regimes [well‐watered (WW), moderate water stress (MWS) and recovery (R)]. Vertical lines indicate the transitions between the three irrigation phases. Values represent means ± SE (*n* = 4). At each time point, different letters indicate significant differences (*p* ≤ 0.05) between treatments according to two‐way ANOVA followed by Duncan's test. *, **, and *** indicate significance at 5%, 1%, and 0.1% probability levels, respectively.

During the second year of experimentation, the degree of graft alignment influenced plant response to water availability (Figure 3). Under WW conditions, all treatments maintained relatively high values of *Ψ*_stem_, *g*_s_, and *A*_N_. Differences were most evident for *A*_N_, with PA alignment in both rootstocks maintaining consistently higher values than other treatments during this initial phase.

During MWS phase, *Ψ*_stem_ progressively decreased in all treatments, reflecting the increasing water deficit. CA plants showed a greater decline in *g*_s_ (0.05 and 0.04 mol H_2_O m^−2^ s^−1^ for 110R and RG8, respectively) compared to PA plants (0.07 and 0.10 mol H_2_O m^−2^ s^−1^ for 110R and RG8, respectively). This reduction in *g*_s_ directly affected *A*_N_, with CA‐110R showing the steepest reduction (1.51 μmol CO_2_ m^−2^ s^−1^), while both alignments in RG8 maintained higher values (5.03 and 3.83 μmol CO_2_ m^−2^ s^−1^ for PA and CA, respectively).

Following rewatering (R phase), all combinations partially recovered *Ψ*_stem_ and *g*_s_, and differences narrowed (Figure 3). However, PA plants, particularly those grafted onto RG8 rootstock, showed a faster recovery compared to CA plants.

**FIGURE 4:**
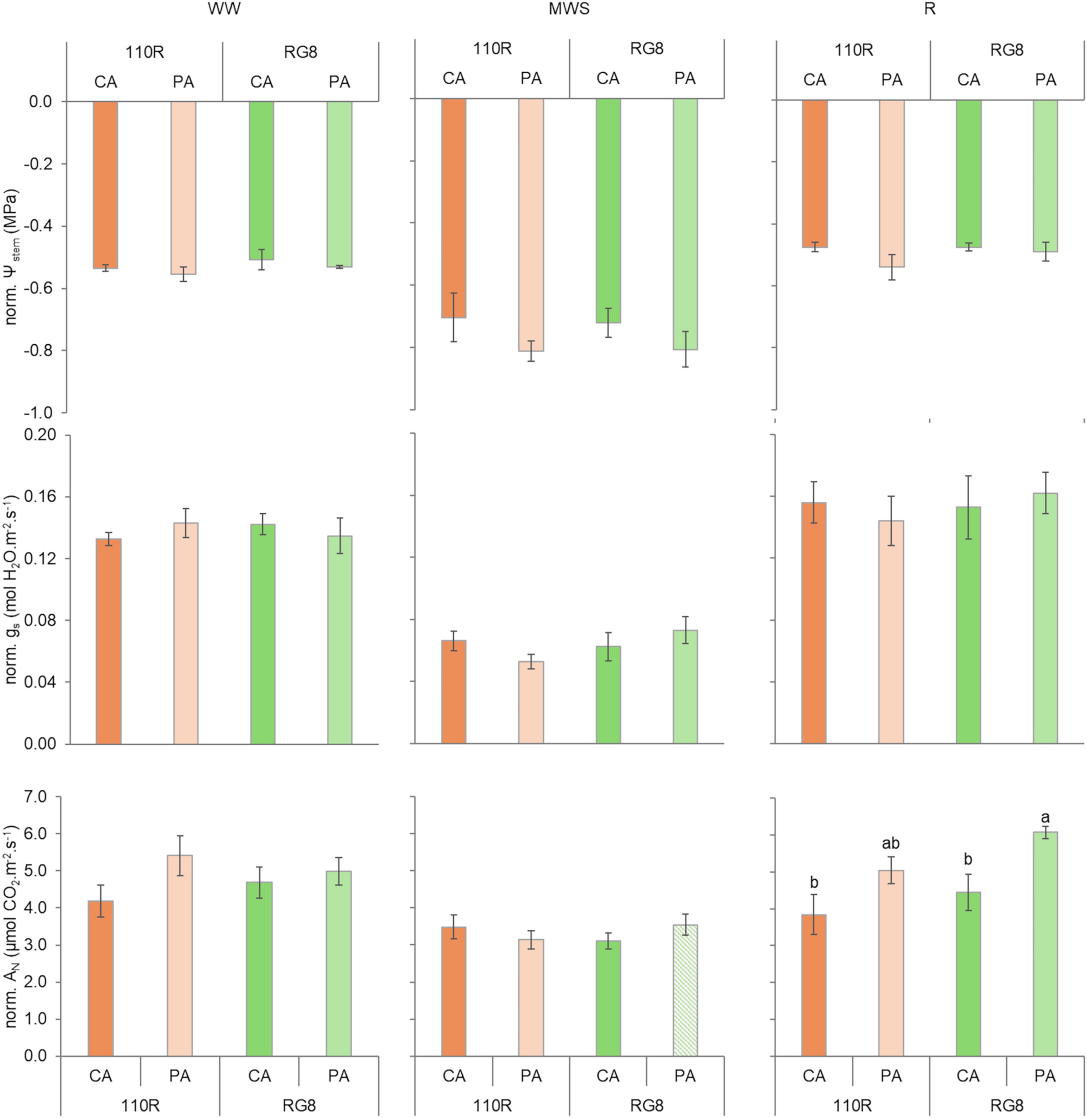
Effect of the rootstock (110R and RG8) and the grafting alignment (CA and PA) on the calculated and normalised AUC of stem water potential (norm. *Ψ*_stem_), leaf stomatal conductance (norm. *g*_s_) and leaf net carbon assimilation (norm. *A*_N_) under three water regimes [well‐watered (WW), moderate water stress (MWS) and recovery (R)]. Values represent means ± SE (*n* = 4). At each time point, different letters indicate significant differences (*p* ≤ 0.05) between treatments according to two‐way ANOVA followed by Duncan's test. *, **, and *** indicate significance at 5%, 1%, and 0.1% probability levels, respectively.

The calculation of the AUC for *Ψ*_stem_, *g*_s_, and *A*_N_ provided a broader view of the physiological response across the three irrigation phases (WW, MWS, and R) (Figure 4). During the WW and MWS phases, all graft alignment–rootstock combinations showed comparable cumulative values. However, during the MWS phase, PA plants tended to exhibit slightly negative *Ψ*_stem_ (−0.81 MPa) compared to CA plants (−0.70 and −0.72 MPa for 110R and RG8, respectively). Despite this trend, no differences were detected in *g*_s_ or *A*_N_.

In the R phase, cumulative *Ψ*_stem_ and *g*_s_ returned to levels comparable to WW conditions, but *A*_N_ exhibited distinct recovery patterns depending on graft alignment and rootstock. PA plants, particularly those grafted onto RG8 rootstock, recovered beyond their initial *A*_N_ values, while CA plants, both rootstocks, showed limited recovery, with *A*_N_ values stabilising at or slightly below their WW levels.

In two‐year‐old plants, differences in vegetative growth parameters were observed depending on the degree of graft alignment, especially in plants grafted onto RG8 rootstock (Table 3). Graft alignment did not affect TCSA, as values were similar between CA and PA plants across both rootstocks. In contrast, SCSA appeared to be affected, with CA plants showing lower shoot development than PA plants, regardless of rootstock (mean CA = 71.4 and mean PA = 98.9 mm^2^). Differences were particularly evident under RG8 rootstock, where CA plants reached 75.3 mm^2^ compared to PA plants 113.7 mm^2^.

**TABLE 3:** Effect of rootstock (R; 110R and RG8) and graft alignment (GA; CA: Completely aligned and PA: Partially aligned) on cumulative trunk and cross‐sectional area (TCSA, SCSA) from 2023 to 2024 (Experiment #2).

Rootstock	Graft alignment	TCSA (cm^2^)	SCSA (cm^2^)
110R	CA	1.55 ± 0.06	67.5 ± 9.7
	PA	1.58 ± 0.09	84.0 ± 8.8
RG8	CA	1.33 ± 0.15	75.3 ± 7.6
	PA	1.64 ± 0.04	113.7 ± 20.7
	R	n.s.	n.s.
	GA	n.s.	**
	R × GA	n.s.	n.s.

*Note:* Mean value (*n* = 4) ± SE. Different letters indicate significant differences (*p* ≤ 0.05) between graft alignment and rootstock according to two‐way ANOVA followed by Duncan's test. n.s., ** indicate non‐significance, significance at 1% probability levels, respectively.

Total plant biomass and its partitioning were mainly influenced by rootstock identity, with graft alignment exerting minor effects (Table 4). The clearest trend was observed in RG8‐PA plants, which accumulated the highest biomass among all combinations. In 110R, CA plants accumulated slightly more total biomass than PA plants (105.2 and 97.7 g, respectively), although both were clearly lower than RG8. Biomass allocation was relatively balanced across alignments, with about one‐third allocated to roots. A trend toward greater root allocation in CA compared with PA plants was detected, reflected in a moderately higher root volume (47.6 and 37.2 cm^3^, respectively).

**TABLE 4:** Effect of the rootstock (110R and RG8) and the grafting alignment (CA and PA) on total plant dry weight (PDW; DW), percentage biomass distribution, root to shoot ratio (R:S), and root volume (RV) after the moderate water stress trial (Experiment #2).

Rootstock	Graft alignment	PDW (*g*)	Biomass distribution (%)			R:S	RV (cm^3^)
			Roots	Rootstock	Scion		
110R	CA	105.2 ± 9.1b	30.2 ± 2.9	58.6 ± 2.5	11.2 ± 0.6	2.7 ± 0.4	47.6 ± 7.4
	PA	97.7 ± 3.3b	25.8 ± 2.1	63.2 ± 2.5	11.0 ± 1.0	2.4 ± 0.3	37.2 ± 2.5
RG8	CA	116.6 ± 8.6b	32.9 ± 3.9	58.0 ± 5.0	9.1 ± 1.1	3.6 ± 0.1	55.5 ± 3.6
	PA	143.6 ± 7.3a	22.4 ± 3.0	70.1 ± 3.5	7.5 ± 0.9	3.0 ± 0.4	47.4 ± 5.6
	R	**	n.s.	n.s.	*	*	n.s.
	GA	n.s.	*	n.s.	n.s.	n.s.	n.s.
	R × GA	*	n.s.	n.s.	n.s.	n.s.	n.s.

*Note:* Mean value (*n* = 4) ± SE. Different letters indicate significant differences (*p* ≤ 0.05) between graft alignment and rootstock according to two‐way ANOVA followed by Duncan's test. n.s., *, ** indicate non‐significance, significance at 5%, and 1% probability levels, respectively.

In RG8, CA plants accumulated intermediate plant weight values (116.6 g), with a biomass allocation similar to 110R‐CA. By contrast, PA plants outperformed all other combinations due to a marked increase in rootstock allocation (70.1% compared to 58% in RG8‐CA plants), while the proportion allocated to roots decreased (22.4% compared to 32.9% in RG8‐CA plants). Despite this shift, root volume remained relatively high.

The only significant graft alignment effect was detected in root allocation, which was greater in CA than PA plants (31.5% and 24.1%, respectively).

### 3.3 Experiment # 3: Comparison of Heterografts, Homografts, and Ungrafted Cuttings

**FIGURE 5:**
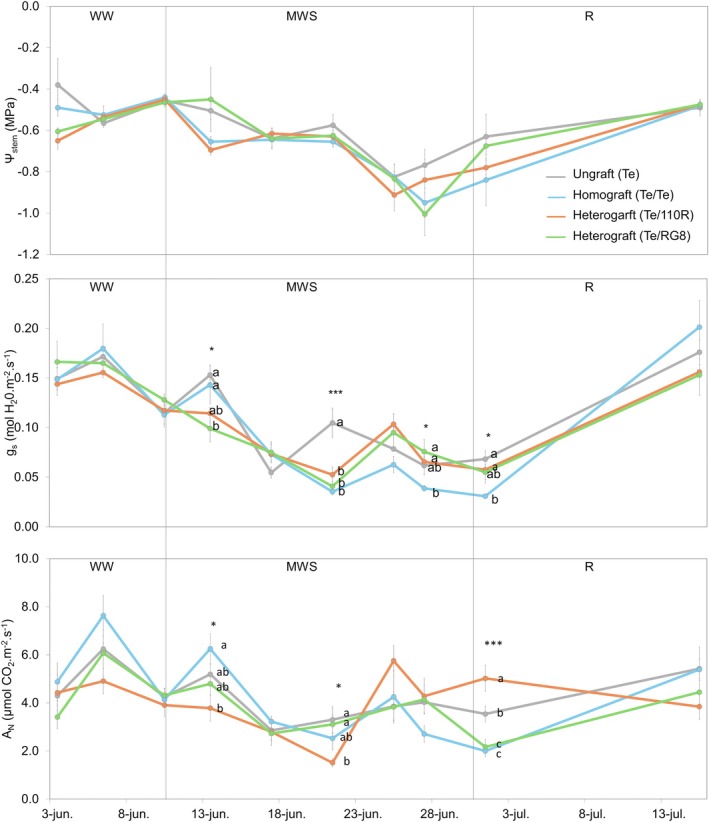
Effect of the partner identity and graft on water potential (*Ψ*_stem_), leaf stomatal conductance (*g*_s_) and leaf net carbon assimilation (*A*_N_) under three water regimes [well‐watered (WW), moderate water stress (MWS) and recovery (R)]. Vertical lines indicate the transitions between the three irrigation phases. Values represent means ± SE (*n* = 4). At each time point, different letters indicate significant differences (*p* ≤ 0.05) between treatments according to one‐way ANOVA followed by Duncan's test. *, **, and *** indicate significance at 5%, 1%, and 0.1% probability levels, respectively.

Water treatments were evaluated across four plant combinations: ungrafted Tempranillo cuttings (Te), CA homografted Tempranillo plants (Te/Te), and CA heterografted Tempranillo onto 110R and RG8 rootstocks (Te/110R and Te/RG8). The four graft combinations modulated *Ψ*_stem_, *g*_s_, and *A*_N_ differently throughout the three irrigation phases (Figure 5). Under WW conditions, grafting and partner identity did not affect plant water status or gas exchange parameters.

During the MWS phase, heterografted plants showed a progressive decline in *g*_s_ (0.1 to 0.05 mol H_2_O m^−2^ s^−1^), whereas homografted and ungrafted plants maintained higher values at the onset of stress (0.15 and 0.14 mol H_2_O m^−2^ s^−1^, respectively). By the end of this phase, *g*_s_ continued to decline in heterografted plants, dropped sharply in homografted plants (0.04 mol H_2_O m^−2^ s^−1^), and remained highest in ungrafted plants (0.1 mol H_2_O m^−2^ s^−1^). *A*_N_ followed a comparable trend; heterografted plants, especially those grafted onto 110R, showed the steepest reduction (3.91 to 1.51 μmol CO_2_ m^−2^ s^−1^), while homografted plants sustained the highest *A*_N_ early in MWS but declined later.

During the R phase, *Ψ*_stem_ recovered to similar levels in all treatments as in WW. However, homografted and ungrafted plants exhibited a faster and greater recovery of *g*_s_ and *A*_N_ compared with heterografted plants.

**FIGURE 6:**
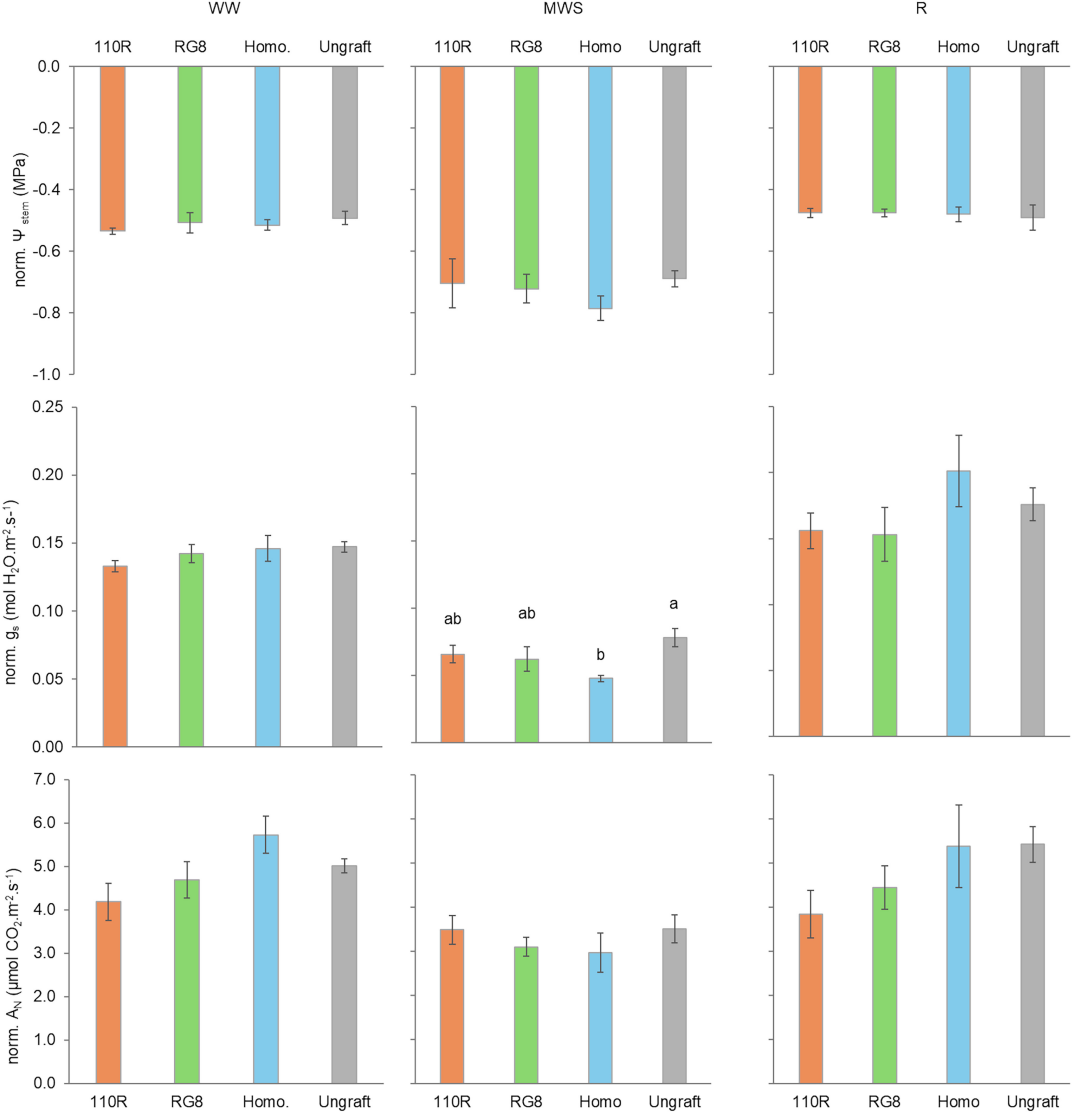
Effect of the partner identity and graft on the calculated and normalised AUC of stem water potential (norm. *Ψ*_stem_), leaf stomatal conductance (norm. *g*_s_) and leaf net carbon assimilation (norm. *A*_N_) under three water regimes [well‐watered (WW), moderate water stress (MWS) and recovery (R)]. Values represent means ± SE (*n* = 4). At each time point, different letters indicate significant differences (*p* ≤ 0.05) between treatments according to one‐way ANOVA followed by Duncan's test. *, **, and *** indicate significance at 5%, 1%, and 0.1% probability levels, respectively. 110R, RG8, Homo and Ungraft represent heterografted, homografted and ungrafted plants, respectively.

Graft combinations modulated plant water status and gas exchange parameters differently across the three irrigation phases (Figure 6). Under WW conditions, no differences were detected among graft combinations. All plants showed comparable values for *Ψ*_stem_ (−0.5 MPa), *g*_s_ (> 0.10 mol H_2_O m^−2^ s^−1^), and *A*_N_ (> 4 μmol CO_2_ m^−2^ s^−1^).

During the MWS phase, differences in physiological response emerged. Ungrafted and heterografted plants maintained higher *g*_s_ values (0.08, 0.07, and 0.06 mol H_2_O m^−2^ s^−1^), while homografted plants showed lower *g*_s_ values (0.05 mol H_2_O‧m^−2^‧s^−1^). A similar pattern was observed for *A*_N_ values; however, no statistically significant differences were found. Ungrafted and heterografted plants reached values above 3 μmol CO_2_ m^−2^ s^−1^, while homografted plants remained below this threshold.

Following rewatering, all graft combinations recovered *Ψ*_stem_ to values similar to the WW phase (−0.5 MPa). Ungrafted and homografted plants showed the highest *g*_s_ (0.18 and 0.2 mol H_2_O m^−2^ s^−1^, respectively) and *A*_N_ (> 5 μmol CO_2_ m^−2^ s^−1^). In contrast, heterografted plants maintained lower *g*_s_ (0.16 mol H_2_O m^−2^ s^−1^) and *A*_N_ during this phase (4.44 and 3.84 μmol CO_2_ m^−2^ s^−1^ for RG8 and 110R, respectively).

The effect of graft combination was significant for both trunk and scion cross‐sectional area (Table 5). For TCSA, grafted plants (both heterografted and homografted) showed significantly greater growth compared to ungrafted plants. No differences were observed between 110R, RG8, and homografted plants, which all maintained similarly high values (> 1.3 cm^2^), while ungrafted vines consistently exhibited the lowest TCSA (1.02 cm^2^).

**TABLE 5:** Effect of the partner identity and graft on cumulative trunk cross‐sectional area (cm^2^) and total shoot cross‐sectional area (cm^2^) (Experiment #3).

Graft combination	TCSA (cm^2^)	SCSA (cm^2^)
110R	1.55 ± 0.06a	67.52 ± 9.68bc
RG8	1.33 ± 0.15a	75.27 ± 8.81b
Homograft	1.44 ± 0.06a	100.41 ± 7.58a
Ungraft	1.02 ± 0.07b	48.25 ± 20.65c
ANOVA	**	***

*Note:* Mean value (*n* = 4) ± SE. Different letters indicate significant differences (*p* ≤ 0.05) according to one‐way ANOVA followed by Duncan's test. ** and *** indicate significance at 1% and 0.1% probability levels, respectively.

A similar pattern was observed for SCSA, where homografted plants achieved the largest cross‐sectional area, exceeding the other graft combinations (100.4 cm^2^). Both heterografted RG8 and 110R maintained intermediate values, whereas ungrafted plants showed the lowest SCSA (75.3, 67.5 and 48.3 cm^2^, respectively).

Total plant biomass was significantly influenced by the graft combination, whereas biomass allocation was not affected (Table 6). Among heterografts, RG8 plants accumulated greater dry weight than 110R, but both remained below homografts. Homografted plants reached the highest biomass overall, while ungrafted plants showed the lowest values (123 and 86 g, respectively).

**TABLE 6:** Effect of the partner identity and graft on total plant dry weight (PDW; DW), percentage biomass distribution, root to shoot ratio (R:S), and root volume (RV) after the moderate water stress trial (Experiment #3).

Graft combination	PDW (*g*)	Biomass distribution (%)			R:S	RV (cm^3^)
		Roots	Rootstock	Scion		
110R	105.2 ± 9.1ab	30.2 ± 2.9	58.6 ± 2.5	30.2 ± 2.9	2.7 ± 0.4b	47.6 ± 7.4b
RG8	116.6 ± 8.6a	32.9 ± 3.9	58.0 ± 5.0	32.9 ± 3.9	3.6 ± 0.1ab	55.5 ± 3.6ab
Homograft	123.8 ± 3.4a	42.0 ± 2.1	47.8 ± 1.8	42.0 ± 2.1	4.2 ± 0.5a	77.4 ± 5.4a
Ungraft	86.9 ± 10.0b	38.9 ± 4.5	48.6 ± 5.4	38.9 ± 4.5	3.2 ± 0.3ab	51.2 ± 10.8b
ANOVA	*	n.s.	n.s.	n.s.	*	*

*Note:* Mean value (*n* = 4) ± SE. Different letters indicate significant differences (*p* ≤ 0.05) according to one‐way ANOVA followed by Duncan's test. n.s. and * indicate non‐significance or significance at 5% probability level, respectively.

Biomass allocation patterns were similar across graft combinations, with no significant differences among roots, rootstock, and scion proportions. However, homografted plants tended to allocate a greater proportion of their biomass to roots (42%) compared with heterografted plants (30% for 110R and 32% for RG8) and ungrafted plants (38%) (Table 6). This allocation was reflected in the root‐to‐shoot ratio, which was highest in homografts, together with their superior root volume. Root volume followed the same trend, with homografts achieving the highest, heterografts intermediate (RG8 > 110R), and ungrafts the lowest values (77.4, 55.5, 47.6 and 51.2 cm^3^, respectively).

## 4 Discussion

The impact of grafting on grapevine growth and physiology can be evaluated through two complementary approaches. The first considers cambial alignment at the graft union, as grafting itself represents a hydraulic constraint (De Herralde et al. 2006; Martínez‐Ballesta et al. 2010), and misalignment is recognised as a critical factor limiting callus bridge development, delaying cambium formation and reducing vascular continuity (Bester 2020). Anatomical and molecular studies have confirmed that successful graft formation requires coordinated cell wall modification, callus proliferation and differentiation of xylem and phloem tissues (Cookson et al. 2013; Clemente Moreno et al. 2014), whereas incomplete alignment results in irregular vascular organisation that may compromise water transport and physiological performance (Milien et al. 2012; Shtein et al. 2017; Marín et al. 2022).

The second approach distinguishes the effect of grafting from that of rootstock–scion interactions by comparing heterografts, homografts, and ungrafted plants. This strategy has shown that homografts exhibit transcriptional and metabolic profiles closer to ungrafted vines, while heterografts display distinct stress‐related signatures (Cookson et al. 2013; Clemente Moreno et al. 2014; Tedesco et al. 2020, 2021, 2023). In addition, scion cultivars and rootstocks have been shown to differ in their vessel characteristics (Battiston et al. 2022; Flor et al. 2025; Gerzon et al. 2015; Santarosa et al. 2016; Shtein et al. 2017), whose interaction may contribute to hydraulic constraints, stomatal regulation, and, ultimately, vine performance under drought.

### 4.1 Graft Alignment Influences Grapevine Growth and Physiology Under Field Conditions

The influence of rootstocks on scion vigour, photosynthesis, and water and nutrient uptake is well established (Buesa et al. 2023; Tramontini et al. 2013; Zhang et al. 2016). However, the impact of graft cambial misalignment between scion and rootstock remains unclear. Marín et al. (2022) reported that growth differences between CA and PA plants diminished after the first year of plantation; nevertheless, our results showed that structural and physiological differences persisted after five growing seasons, suggesting that graft alignment can affect plant performance beyond establishment (Figure 2 and Table 2). These discrepancies may be explained by climatic conditions; thus, years assessed by Marín et al. (2022) were cooler and wetter than the decadal mean, whereas our experimental period included more extreme conditions, with one of the hottest and driest seasons of the last decade (Figure S1), which may have amplified the mid‐term effects of graft alignment on growth.

CA plants, particularly on 110R, maintained a more favourable water status (Medrano 2002) and higher *A*_N_ compared to PA plants, which explained their superior SCSA (Tables 1 and 2). These findings support the hypothesis that proper vascular continuity at the graft union may reduce hydraulic resistance and improve physiological and growth performance (Adams et al. 2018; Battiston et al. 2022; Korkutal et al. 2018; Milien et al. 2012). By contrast, PA plants generally performed worse than CA plants, although under RG8, they maintained similar or even better physiological activity. As shown by Camboué et al. (2025), omega grafts distributed irregularly functional xylem vessels around the graft union, with enhanced connections often appearing below the bud. It is plausible that in PA plants, this vascular pathway, together with the high inherent vigour of RG8 (Marín et al. 2023; Buesa et al. 2023), likely compensated for misalignment, maintaining sufficient xylem connectivity to support growth.

Despite the differences observed between CA and PA plants, yield and berry composition remained unchanged during the study period (Table S1). In long‐term field studies, increases in photosynthesis and water status translated into higher yield in Tempranillo (Medrano et al. 2003). Similarly, Torres et al. (2021) reported in Cabernet Sauvignon a strong correlation between seasonal water status, gas exchange, and yield. In our vineyard, CA and PA plants exhibited moderate physiological differences, which appear insufficient to modify reproductive traits within the evaluated period.

### 4.2 Graft Alignment Modulates Drought Responses in Young‐Grafted Plants

CA plants exhibited a conservative behaviour under moderate water stress, with an earlier decline in *g*_s_ and greater biomass allocation to roots, consistent with a drought‐avoidant strategy (Sperry et al. 2002). In contrast, PA plants sustained higher *g*_s_, particularly on RG8, and recovered *A*_N_ faster after rewatering (Figures 3 and 4), reflecting a drought‐tolerant behaviour (Bartlett et al. 2012; Schultz 2003), considered advantageous under mild drought (McDowell et al. 2008), and associated with improved recovery (Pou et al. 2012). In line with this response, PA plants invested more biomass into the shoot and rootstock rather than into roots, supporting higher SCSA (Tables 3 and 4), a mechanism that resembles the response described by Serra et al. (2014), where new absorbing roots produced under stress increased water uptake and growth. Such growth patterns could reflect the fact that partial graft misalignment may impair phloem continuity (Milien et al. 2012), potentially favouring assimilate retention in the scion, as observed under girdling treatments (Hunter and Ruffner 2001), thereby contributing to increased shoot growth while limiting carbon supply to the root system (Tables 3 and 4).

These contrasting strategies may be related to differences in hydraulic connectivity at the graft union. Grapevine cultivars have been shown to differ in susceptibility to stomatal regulation under water deficit due to intrinsic hydraulic traits (Dayer et al. 2020; Tombesi et al. 2014, 2015). In omega grafts, structural differences in vessel production and continuity have also been reported, with well‐connected unions displaying organised vascular structures, while poorly connected unions showed greater vessel discontinuity and irregular wood density (Milien et al. 2012). Such variability in vascular organisation could have influenced the efficiency of chemical and hydraulic signalling involved in stomatal regulation (Soar et al. 2006), since, as observed by Villa‐Llop et al. (2022), CA omega grafts have higher hydraulic conductance than PA grafts, indicating reduced water‐transport efficiency in misaligned unions.

### 4.3 Grafting and Rootstock‐Scion Interaction Modulated Physiological Responses and Growth in Young‐Grafted Plants Under Drought

Under WW conditions, neither the presence of a graft union nor the identity of the partner compromised plant water relations or gas exchange (Figures 5 and 6), consistent with previous reports that show grafting does not constrain water status under non‐limiting conditions (Iacono et al. 1998).

In contrast, under MWS, ungrafted plants maintained higher physiological performance than grafted plants, which exhibited overall lower *g*_s_ values (Figure 6). This suggests that the graft union itself may represent a hydraulic discontinuity that increases sensitivity to water deficit, as reported in earlier studies (Atkinson et al. 2003; Bavaresco and Lovisolo 2000; De Herralde et al. 2006; Torii et al. 1992) that found reduced hydraulic conductance in grafted plants.

Despite these physiological limitations, grafting promoted growth, with homografts achieving the greatest values (Table 5). This was likely due to the more developed root systems produced during nursery propagation, which enhanced water and nutrient uptake capacity compared to ungrafted plants. Homografted plants also exhibited higher root‐to‐shoot ratios and greater root volume than heterografted plants (Table 6). These differences could be partially explained by genotype‐specific xylem traits. Tempranillo cv. was reported to display a greater density of narrower xylem vessels compared to 110R, potentially increasing the total vessel surface area and thereby enhancing hydraulic conductance on the whole plant (Camboué et al. 2025; Flor et al. 2025; Lamarque et al. 2023; Ramsing et al. 2021). Although no anatomical data are available for RG8, its vessel characteristics may resemble those of 110R, given their shared parentage. In addition, Tedesco et al. (2021) demonstrated that *V. vinifera* homografts displayed more efficient phloem translocation compared to heterografts. Consequently, the combination of greater xylem conductive area and enhanced phloem transport in Tempranillo homografts may support the improved root and shoot growth observed.

## 5 Conclusions

The complementary approaches assessed in the present study provided new evidence that cambial alignment, grafting, and partner interactions affected plant development and physiological performance. Completely aligned grafts enhanced vegetative development and gas exchange. The vigorous RG8 rootstock further mitigated the impact of misalignment, maintaining comparable or even improved physiological performance in PA plants. Grafting itself increased plant sensitivity to moderate water stress, while greater genotype affinity promoted higher biomass allocation to both shoots and roots. Further long‐term studies are required to investigate whether differences in vascular connectivity at the graft union affect xylem and phloem functionality, hormone transport, or plant response to drought, and whether these structural and physiological traits influence vine longevity under different scion‐rootstock combinations.

## Author Contributions

A.V.‐L., I.B., M.V, M.L., A.S. and N.T. carried out experiments. A.V.‐L. carried out the statistical analyses. A.V.‐L., I.G., L.G.S. and N.T. contributed to data interpretation. A.V.‐L., J.M.E., I.B., L.G.S. and N.T. conceived the study, designed the experiment, and coordinated all research activities. A.V.‐L. wrote the manuscript. All the authors revised and approved the final manuscript.

## Funding

The research was carried out within the project PID2021‐123305OB‐C32 (UPGRAPE) funded by MICIU/AEI/10.13039/501100011033 and by the European Union Next Generation EU/PRTR and by FEDER, UE and of VITES QUALITAS (EFA 324/19) and VITRES (EFA 033/01), projects co‐funded at 65% by the European Regional Development Fund (ERDF) through the Interreg Program V‐A Spain‐France‐Andorra (POCTEFA 2014–2020) and the Interreg Program VI‐A Spain‐France‐Andorra (POCTEFA 2021–2027). The aim of POCTEFA is to reinforce economic and social integration in the Spain‐France‐Andorra border area. A. Villa‐Llop acknowledges the funding for an Industrial pre‐doctoral contract of the Government of Navarra (Ref. 283E/2020). M. Velaz acknowledges the funding for a predoctoral contract from the Universidad Pública de Navarra (RES 2640/2023). I. Buesa is grateful for funding from the Generalitat Valenciana, Plan GenT [CIDEIG/2023/7]. N. Torres thanks the funding of a Ramón y Cajal Grant (RYC2021‐034586‐I) funded by MCIN/AEI/10.13039/501100011033 and by ‘European Union Next Generation EU/PRTR’.

## Supporting information


**Figure S1:** Monthly rainfall and accumulated GDD from April to October of the mean past decade (2013–2023), 2021, 2022 and 2023 seasons, in Murieta, Navarra, Spain. Data obtained from https://servicio.mapa.gob.es/websiar/.
**Table S1:** Effect of rootstock (R; 110R and RG8) and graft alignment (GA; CA: completely aligned and PA: partially aligned) on yield component and berry composition [Yield (Y), number of clusters per plant (No C), mean cluster weight (CW), berry weight (BW), potential alcohol (PA) and pH].
**Table S2:** Effect of rootstock (R; 110R and RG8) and graft alignment (GA; CA: completely aligned and PA: partially aligned) on stomatal conductance (gs), net photosynthesis (AN), evapotranspiration (E) and vapour pressure deficit (VPD), measured in 2023.

## Data Availability

The data that support the findings of this study are available from the corresponding author upon reasonable request.
