# Supplementary material for: Exploring the Impact of Vascular Alignment and Grafting on Grapevine Physiology and Growth
Source: Physiol Plant. 2025 Dec 28;178(1):e70704. doi: 10.1111/ppl.70704 (PMC12745673; doi:10.1111/ppl.70704)
Supplement: Supplementary file 1 — Figure S1: Monthly rainfall and accumulated GDD from April to October of the mean past decade (2013–2023), 2021, 2022 and 2023 seasons, in Murieta, Navarra, Spain. Data obtained from https://servicio.mapa.gob.es/websiar/. Table S1: Effect of rootstock (R; 110R and RG8) and graft alignment (GA; CA: completely aligned and PA: partially aligned) on yield component and berry composition [Yield (Y), number of clusters per plant (No C), mean cluster weight (CW), berry weight (BW), potential alcohol (PA) and pH]. Table S2: Effect of rootstock (R; 110R and RG8) and graft alignment (GA; CA: completely aligned and PA: partially aligned) on stomatal conductance (gs), net photosynthesis (AN), evapotranspiration (E) and vapour pressure deficit (VPD), measured in 2023. [file PPL-178-e70704-s001.pdf]

Supplementary material

Exploring the impact of vascular alignment and grafting on grapevine physiology and growth

Ana Villa-Llop[1,2,3*], Ignacio Buesa[4,5], Maider Velaz[2,3], Maite Loidi[3], José Mariano Escalona[5,6], Antoni Sabater[6], Luis Gonzaga Santesteban[2,3] and Nazareth Torres[2,3]

Supplementary Figures

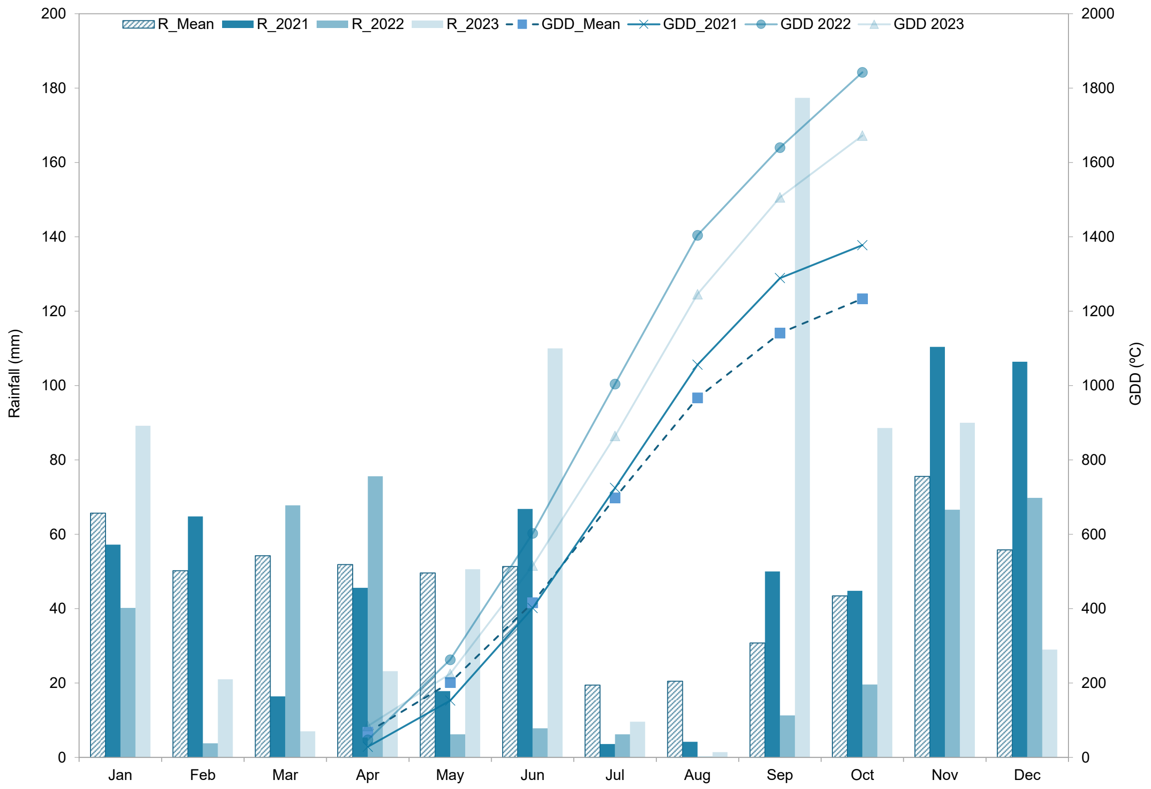

Figure S1. Monthly rainfall and accumulated GDD from April to October of the mean past decade (2013-2023), 2021, 2022 and 2023 seasons, in Murieta, Navarra, Spain. Data obtained from https://servicio.mapa.gob.es/websiar/

Supplementary Tables

Table S1. Effect of rootstock (R; 110R and RG8) and graft alignment (GA; CA: completely aligned and PA: partially aligned) on yield component and berry composition [Yield (Y), number of clusters per plant (No C), mean cluster weight (CW), berry weight (BW), potential alcohol (PA) and pH].

	Rootstock	Graft alignment	Y (kg)	No C	CW (g)	BW (g)	PA (º)	pH
2021	110R	CA	1.5 ± 0.1	10.3 ± 0.5	148.4 ± 6.5	1.7 ± 0.1	14.5 ± 0.6	3.64 ± 0.02
		PA	1.6 ± 0.2	9.93 ± 1.4	161.1 ± 4.6	1.7 ± 0.1	13.9 ± 0.5	3.64 ± 0.06
	RG8	CA	1.4 ± 0.1	10.8 ± 1.3	133.1 ± 6.4	1.6 ± 0.1	13.8 ± 0.4	3.68 ± 0.02
		PA	1.9 ± 0.2	12.2 ± 0.8	154.4 ± 13.4	1.6 ± 0.0	14.0 ± 0.3	3.75 ± 0.01
		R	*n.s.*	*n.s.*	*n.s.*	*n.s.*	*n.s.*	*n.s.*
		GA	*n.s.*	*n.s.*	*n.s.*	*n.s.*	*n.s.*	*n.s.*
		R × GA	*n.s.*	*n.s.*	*n.s.*	*n.s.*	*n.s.*	*n.s.*
2022	110R	CA	3.2 ± 0.3	16.2 ± 1.3	194.1 ± 11.7	2.0 ± 0.0	13.3 ± 0.1	3.48 ± 0.02
		PA	3.3 ± 0.6	16.6 ± 1.2	196.7 ± 26.5	1.7 ± 0.1	13.4 ± 0.4	3.54 ± 0.02
	RG8	CA	3.4 ± 0.5	16.2 ± 2.1	218.9 ± 41.6	1.7 ± 0.2	12.9 ± 0.3	3.54 ± 0.02
		PA	4.4 ± 0.9	18.7 ± 2.6	232.2 ± 24.8	2.0 ± 0.2	13.5 ± 0.3	3.54 ± 0.04
		R	*n.s.*	*n.s.*	*n.s.*	*n.s.*	*n.s.*	*n.s.*
		GA	*n.s.*	*n.s.*	*n.s.*	*n.s.*	*n.s.*	*n.s.*
		R × GA	*n.s.*	*n.s.*	*n.s.*	*n.s.*	*n.s.*	*n.s.*
2023	110R	CA	1.3 ± 0.3	11.9 ± 2.2	103.6 ± 8.6	2.0 ± 0.1 Y	13.4 ± 0.4	3.42 ± 0.02
		PA	1.3 ± 0.5	11.5 ± 2.8	106.9 ± 17.4	1.8 ± 0.1 Y	12.9 ± 0.6	3.49 ± 0.05
	RG8	CA	1.6 ± 0.4	13.8 ± 1.7	110.1 ± 15.1	1.5 ± 0.2 Z	12.7 ± 0.3	3.54 ± 0.08
		PA	1.9 ± 0.6	13.9 ± 3.1	127.1 ± 18.6	1.7 ± 0.1 Z	13.4 ± 0.5	3.46 ± 0.04
		R	*n.s.*	*n.s.*	*n.s.*	*	*n.s.*	*n.s.*
		GA	*n.s.*	*n.s.*	*n.s.*	*n.s.*	*n.s.*	*n.s.*
		R × GA	*n.s.*	*n.s.*	*n.s.*	*n.s.*	*n.s.*	*n.s.*

Mean values (n = 3) ± SE. Uppercase letters indicate significant differences ($P \leq 0.05$) for rootstock (R) according to two-way ANOVA followed by Duncan's post-hoc test. n.s. and * indicate non-significant and significance at 5% probability levels, respectively.

Table S2. Effect of rootstock (R; 110R and RG8) and graft alignment (GA; CA: completely aligned and PA: partially aligned) on stomatal conductance (g_s), net photosynthesis (A_N), evapotranspiration (E) and vapour pressure deficit (VPD), measured in 2023.

Rootstock	Graft alignment	Ψ_{pd} (MPa)	Ψ_{stem} (MPa)	g_s (mol $H_2O.m^{-2}.s^{-1}$)	A_N (µmol $CO_2.m^{-2}.s^{-1}$)
110R	CA	-0.28 ± 0.02	-0.57 ± 0.05	0.29 ± 0.01	8.97 ± 1.28
	PA	-0.26 ± 0.04	-0.49 ± 0.06	0.30 ± 0.04	7.79 ± 1.24
RG8	CA	-0.25 ± 0.05	-0.46 ± 0.05	0.24 ± 0.02	6.44 ± 0.72
	PA	-0.18 ± 0.05	-0.42 ± 0.01	0.33 ± 0.01	6.92 ± 0.81
	R	*n.s.*	*n.s.*	*n.s.*	*n.s.*
	GA	*n.s.*	*n.s.*	*n.s.*	*n.s.*
	R × GA	*n.s.*	*n.s.*	*n.s.*	*n.s.*
